# Effect of the BMPR-II-SMAD3/MRTF pathway on proliferation and migration of ASMCs and the mechanism in asthma

**DOI:** 10.1007/s11033-022-07764-9

**Published:** 2022-08-25

**Authors:** Wenbo Gu, Jiahui Lei, He Zhu, Yali Xiao, Zhenping Zhang, Limin Zhao

**Affiliations:** 1 Henan University of Chinese Medicine, Zhengzhou, China; 2 Zhengzhou University People’s Hospital, Zhengzhou, China; 3 People’s Hospital of Zhongmu, Zhengzhou, China; 4 Henan Provincial People’s Hospital, Zhengzhou, China; 5 Henan Univerity People’s Hospital, Zhengzhou, China; 6 Department of Respiratory Medicine, Henan Provincial People’s Hospital (Zhengzhou University People’s Hospital), No. 7 Weiwu Road, Zhengzhou, Henan China

**Keywords:** Asthma, ASMCs, BMPR-II-SMAD3/MRTF complex, Ca^2+^ concentration, Proliferation, Migration

## Abstract

**Background:**

A variety of smooth muscle-specific genes and proteins, including SMAD3, BMPR-II, and MRTF, are involved in airway remodeling in asthma. As a receptor of bone morphogenetic protein (BMP) signaling, BMPR-II has important roles in airway remodeling in asthma. However, the underlying mechanism of BMPR-II in airway smooth muscle cells (ASMCs) in asthma remains incomplete.

**Methods:**

Wistar rats were intraperitoneally injected with ovalbumin antigen suspension and aluminium hydroxide and, stimulated with ovalbumin nebulized inhalation to constructed asthma model. Primary ASMCs were isolated with collagenase I and identified by testing the α-SMA expression. Quantitative polymerase chain reaction (qPCR) and western blot assay were employed to detect the gene expression. CCK8, Transwell and Fluo-4 A assays were introduced to measure the cell viability, migration and intracellular Ca^2+^. Co-Immunoprecipitation (Co-IP) assay was applied to test the interaction among proteins.

**Results:**

First, we observed significant increases in BMPR-II in asthmatic rat model and ASMCs at both the mRNA and protein levels. Second, we observed that silencing of siBMPR-II inhibited proliferation, migratory capacity and intracellular Ca^2+^ concentration in ASMCs. Furthermore, our study demonstrated that siBMPR-II inhibited the Smad3 expression and overexpression promoted the bioactivity of ASMCs. In addition, this study showed that p-Smad3 could interacted with MRTF and siMRTF inhibits the bioactivity of ASMCs. Finally, our results revealed BMPR-II-SMAD3/MRTF pathway affected the bioactivity of ASMCs.

**Conclusions:**

This study indicates that the BMPR-II-SMAD3/MRTF signaling pathway is involved in the process of ASMCs remodeling, providing novel avenues for the identification of new therapeutic modalities.

## Introduction

Asthma is a common chronic respiratory disease that poses a serious potential threat to patients’ physical and mental health [1]. Symptoms of asthma attacks primarily include chest tightness, wheezing, short of breath, and coughing. These symptoms are closely correlated with variable airflow limitation, airway inflammation, airway hyperresponsiveness, and airway remodeling [2]. Previous studies have claimed that airway inflammation is a prerequisite of airway remodeling [3]. However, this view has been recently debated by some scholars who posit that airway remodeling may occur prior to airway inflammation and may be the leading cause of asthma attacks [3]. Studies have shown that overexpression of ORMDL3 may induce abnormal proliferation and contractility of ASMCs in the absence of airway inflammation [4]. This research further confirmed that abnormal expression of specific gene and proteins in ASMCs may be the primary event in asthma rather than a secondary response to airway inflammation. Clonal findings have verified that anti-inflammatory therapy, while improving the symptoms and controlling acute episodes, does not change the nature of airway remodeling. Therefore, many patients experience refractory asthma [5]. The results above all confirmed that airway remodeling plays a pivotal role in the pathogenesis of asthma. To overcome this disease, it is essential to identify an effective therapy to reverse airway remodeling.

Airway remodeling refers to changes in structure and function of the airway wall [6]. Pathological airway remodeling primarily includes epithelium shedding, subepithelial fibrosis, goblet cell hyperplasia, and ASMC hyperplasia and hypertrophy [6]. Recently, increasing research has found that the mass of the ASM layer in asthmatic airway tissue is significantly thicker than that in non-asthmatics [7]. Biopsies from patients with severe asthma reveal larger ASM areas and shorter distance between the subepithelial basement membrane and airway smooth muscle. Both ASM zone and SBM-ASM distance are closely related to FEV1 before and after use of bronchodilators in asthma patients, indicating that ASM content may be closely related to the severity of airway obstruction and may represent one of the critical determinants of disease severity [8]. The increase in ASM mass is classically defined as increased cell numbers resulting from augmented proliferation and decreased apoptosis, as well as cellular hypertrophy. Meanwhile, ASMCs become hypertrophic and other cells differentiate into ASMCs and migrate to into the ASM bundle, which also contributes significantly to augmented ASM content [9]. Nevertheless, the specific mechanisms mediating the bioactivity of ASMCs remain poorly understood.

Recently, several reports in the literature have correlated certain signaling molecules, include SMAD3, BMPR-II, and MRTF, to asthma progression. SMAD3 dependent TGF-β1 signaling has been validated to play a predominant role in airway remodeling in asthma [10]. Wang Meng et al. discovered that Rhynchophylline (RHY) inhibits aberrant ASMC proliferation and alleviates airway inflammation, as well as allergic symptoms, by blocking TGF-β1-mediated Smads and MAPK signaling [11]. Previous studies indicated that BMP signaling plays a critical role in normal lung development [12]. BMP ligand signals are transmitted downstream by phosphorylating R-Smads, including Smad2 and Smad3, and are involved in modulating gene expression in the nucleus [13, 14]. Harsha et al’s research showed lower expression of BMPR-II on airway epithelium in mild asthma [12]. However, expression patterns on ASMCs in asthma patients remain unclear.

Several reports have previously demonstrated that TGF-β1 promotes MRTF nuclear translocation through a WNT-11 dependent mechanism and accelerates expression of smooth-α-actin through the Rho-actin-MRTF axis, further aggravating ASM remodeling [15]. During the progress of lens epithelial-mesenchymal transformation (EMT), Smad3 inhibitor treatment restrained MRTF-A nuclear translocation induced by TGF-β and reduced expression of α-SMA, revealing that Smad3 is an upstream regulator of MRTF-A nuclear translocation and that the two together regulate expression of α-SMA [16]. However, Previous study found that loss of functional BMPR-II induces loss of SMAD3, which directly promotes the proliferation and migration of pulmonary arterial smooth muscle cells (PASMCs) and indirectly induces hypertrophy of PASMCs by augmenting α-SMA expression via MRTF-dependent signaling [17]. Taken together, we concluded that the interactions among BMPR-II, SMAD3, and MRTF participate in regulating the bioactivity of various cell types. Moreover, our preliminary experiments demonstrated high expression of BMPR-II, SMAD3, and MRTF in ASMCs. Nevertheless, it remains unclear how these three factors interact and whether they are involved in regulating the bioactivity of ASMCs.

The aim of the study was to investigate the role of BMPR-II-SMAD3/MRTF pathway in ovalbumin-induced asthmatic rats, and to explore whether an upstream and downstream relationship exists among these three factors in primary ASMCs.

## Materials and methods

### Preparation of asthma model

All animal experiments were conducted in accordance with the International Animal Ethics Committee and the Animal Ethics Guidelines of Henan University of Traditional Chinese Medicine. The animal experiment passed the review of Ethics Committee on Laboratory Animals of the First Affiliated Hospital of Henan University of Traditional Chinese Medicine, the experimental animal ethical review approval number is YFYDW2019032. Twenty SPF Wistar rats (body weight 200 ± 20 g, half male and female) were provided by the Animal Experiment Center of Henan University of Traditional Chinese Medicine. Rats were kept in sterile cages at a constant temperature of 20–25 °C and a relative humidity of 50–70%. All rats were provided filtered water and pellet feed *ad libitum*. Twenty rats were randomly assigned to either the control or asthma group with 10 rats per group.

The asthma rat model was constructed as previous study described [18]. In brief, rats were intraperitoneally injected with 100 µg ovalbumin antigen suspension (Sigma-Aldrich, USA) and 1 mg aluminium hydroxide (Sigma-Aldrich, USA) on day 1, 7, and 15. Starting on the 21st day of the experiment, rats were placed in a closed container and stimulated with 5% OVA nebulized inhalation, for 30 min each, 1 ml/min, once/day for 6 weeks. The normal control group was intraperitoneally injected with the same amount of normal saline on the same schedule as the asthma group, and the same amount of normal saline was used for atomization.

### Isolation of primary ASMCs

Within 24 h after the end of the last nebulization challenge, rats were anesthetized and sacrificed by intraperitoneal injection of 20% urethane at a dose of 5 ml/kg. Part of the trachea was obtained and fixed in 4% paraformaldehyde for haematoxylin-eosin staining (HE) and analysis (ServiceBio, Wuhan, China), and histological images were used to analyse pathophysiological changes in tracheal tissue structure in asthmatic rats under ×200 magnification.

The remaining tissue samples were used for isolation of primary ASMCs. Briefly, after removing the connective tissue and fat around the trachea under a microscope, tissues were cut into 1 mm × 1 mm × 1 mm pieces, digested with collagenase I at 37 ℃ for 1 h, and centrifuged at 200 rpm for 5 min. Then, pellets were placed in complete DMEM/F-12 (Junxin biotech, Suzhou, China) containing 10% foetal bovine serum (FBS, Gibco, USA), 100 unit/ml penicillin and 100 mg/ml streptomycin (FBS, Gibco, USA) in an incubator containing 5% CO2 at 37 °C. Media were changed every 3 days until the cell confluence reached 90%, and then cells were passaged using 0.25% trypsin-EDTA solution and used for experiments within 3–8 generations. Hematoxylin-eosin (HE) staining was performed by Wuhan Servicebio Biological Technology Co., Ltd. Non-invasive monitoring of respiratory parameters in rats was performed through WBP whole body plethysmography system (Tawang Intelligent Shanghai, China).

### Immunofluorescence assay

After their respective treatments, cells were washed with PBS three times and fixed in pre-cooled 4% paraformaldehyde (PFA) at room temperature (RT) for 30 min and permeabilised with 0.05% Triton at RT for 5 min. Subsequently, cells were blocked in 1% BSA for 1 h at RT. Then, cells were incubated in anti-α-SMA antibody (Abcam, Cambridge, UK) overnight at 4 ℃ followed by incubation with goat anti-rabbit antibody (Abcam, USA) at RT for 1 h. After staining with Hoechst (Junxin biotech, Suzhou, China) at 37 ℃ for 5–10 min, coverslips fluorescent sealant were applied to slides, which were then examined using a microscope (Olympus, Japan).

### Cell viability assays

Cell proliferation was measured using the Cell Counting Kit-8 assay (CCK8, Junxin, Suzhou, China) according to the manufacturer’s instructions. Cells were plated into 96-well plates at a concentration of 0.2 × 10^5^ cells/well. After their respective treatments, 10 µl CCK8 agents were added into each well, and cells were incubated for 2 h followed by measurement of the absorbance 450 nm using a microplate reader (Thermo).

### Cell proliferation assays

Cells were plated into 48-well plates at a concentration of 0.5 × 10^5^ cells/well. After their respective treatments, 200 µl EdU (Junxin, Suzhou, China) agents were added into each well, and cells were incubated for 2 h followed by operations according to the manufacture’s instructions.

### Transwell assays

Cell migration ability was tested in Transwell chambers (Corning, USA). ASMCs were digested using 0.25% trypsin, 400 µl serum culture medium was added to Transwell chambers of a 24-well plate, and cells at a concentration of 2 × 10^5^/ml in serum-free medium were added to Transwell chamber inner chambers in 100 µl. Next, 600 µl DMEM/F-12 media containing 10% FBS was placed into each outer chamber as a chemoattractant. After incubation at 37 °C for 24 h, non-migrated cells on the upper surface of the filter were removed with a cotton swab. After cells that were adhered to the lower surface of the filter were fixed in paraformaldehyde, 0.1% crystal violet stain was applied for 20 min. Finally, the number of stained cells was counted under a microscope.

### RNA extraction and quantitative RT-PCR

Total RNA was extracted from ASMCs using an RNA extraction kit (Junxin, Suzhou, China). cDNA was isolated according to the instructions of the cDNA synthesis kit (Invitrogen, Carlsbad, CA). Real-time quantitative PCR (qPCR) was performed using SYBR Select Master Mix (Junxin, Suzhou, China). PCR reaction conditions were as follows: 95 °C pre-denaturation for 10 min, followed by 40 denaturation cycles, 95 °C denaturation for 10 seconds, and 60 °C denaturation for 30 seconds. PCR amplification products were analysed on an ABI 7900 fast thermal cycler (Applied Biosystems, ABI). β-actin was measured as a control. Data were calculated using the 2^−ΔΔCt^ method. Primers used in this study were synthesized by Sangon Biotech (Shanghai, China) and listed below: BMPR-II (forward primer): 5′-TCCGGGCAGGATAAATCAGGA-3′, and BMPR-II (reverse primer); 5′-GATTCTGGGAAGCAGCCGTA-3′. SMAD3 (forward primer): 5′-CGCATGAGCTTCGTCAAAGG-3′, and SMAD3 (reverse primer); 5′-CCGATCCCTTTACTCCCAGTG-3′. MRTF (forward primer): 5′-CTTTCTCAGCTCCCAATGGCT-3′, and MRTF (reverse primer); 5′-ACTTCTCGCTCGCAGACTTC-3′. β-actin (forward primer): 5′-ATGGATGACGATATCGCTGC-3’, and β-actin (reverse primer); 5′-CTTCTGACCCATACCCACCA-3′.

### Western blot analysis

ASMC protein was isolated using RIPA buffer (Sangon biotech, Shanghai, China), and the protein concentration was determined using the BCA method (Yeasen Biotech, Shanghai, China) as previous paper described [19]. Thirty micrograms of protein per well were run on 10% SDS-polyacrylamide (sodium dodecyl sulphate polyacrylamide) gel electrophoresis and then transferred to PVDF membranes (Millipore, USA). Blots were blocked in 5% bovine serum albumin at room temperature for 1 h and incubated with primary antibodies overnight at 4 ℃ followed by incubation with corresponding secondary antibodies at room temperature for 1 h. Then, membranes were exposed to ECL reagent (Junxin, Suzhou, China). β-actin was measured as a loading control. Antibodies used in this study are listed below: anti-BMPR-II antibody (Abcam 130206), anti-SMAD3 antibody (Abcam 208182), anti-pSMAD3 antibody (Abcam 118825), anti-MRTF antibody (Abcam 49311) and anti-β-actin antibody (Abcam 8226) was obtained from Abcam (Cambridge, UK) and operated according to the manufacturer’s instructions.

### Cell transfection

Small interfering RNA (siRNA) was designed based on the known cDNA sequences of BMPR-II and MRTF D in the rat gene library and synthesized by GenePharma (Shanghai, China). The sequences were listed below: siBMPR-II-1: 5′-GACGCAUGGAGUAUUUGCUUGUGAU-3′; siBMPR-II-2: 5′-CAGCUGACAGAAGAAGACUUGGAAA-3′; siBMPR-II-3: 5′-CAGUCCUGAUGAACAUGAACCUUUA-3′; siMRTF-1: 5′-GCACAUGGAUGAUCUGUUUTT-3′; siMRTF-2: 5′-GCCUCCGUUAACACCACAATT-3′; siMRTF-3: 5′-GGUAUUUAUUCAAAGUCCAUCAAAU-3′; siNC: 5′-UUCUCCGAACGUGUCACGUTT-3′. SMAD3 overexpression vectors were constructed in Suzhou Junxin Biotechnology Co., Ltd (Suzhou, China).

siBMPR-II, siMRTF, siNC, pcDNA-SMAD3 and pcDNA- NC were transfected into ASMCs using Lipofectamine 3000 (Invitrogen, USA) according to the manufacturer’s protocol. About 48 h after transfection, cells were harvested for further analysis.

### Detection of intracellular Ca^2+^ fluorescence by laser confocal microscopy

Intracellular Ca^2+^ concentrations were detected according to the instructions of the green fluorescent indicator Fluo-4AM (Yeasen Biotech, Shanghai, China) protocol. Briefly, ASMCs were plated on coverslips and used for detection after 48 h of growth. Before detection, cells were treated with 4 µM Fluo4-AM (Sigma, Japan) in serum-free media and incubated at 37 °C for 30 min. The blank group was treated with addition of a corresponding volume of D-Hank’s solution. Results were observed under an Olympus IX-70 laser confocal microscope (Olympus, Japan).

### Co-immunoprecipitation (Co-IP)

Co-IP assays were performed using a Co-Immunoprecipitation kit (Thermofisher, USA) according to the manufacturer’s instructions. Briefly, cells were rinsed twice in ice-cold PBS and then placed in 1 ml pre-chilled lysis buffer and incubated with 100 µl protein A/G beads (Biolinkedin, Shanghai, China) at 4 ℃ for 15 min to eliminate non-specific interactions. Then, 500 µl of lysate was incubated with 5 µg of MRTF and p-Smad3 antibodies and rotated at 4 ℃ overnight. After washing and elution, precipitated proteins were measured by western blot analysis.

### Statistical method

SPSS 15.0 software (SPSS, Inc., Chicago, IL, USA) was used to analyse the data, which are presented as the means ± SEM. Data were analysed with t tests and one-way analysis of variance. Statistical significance was defined as P < 0.05.

## Results

### BMPR-II were upregulated in the airway of allergic asthma rats

**Fig. 1:**
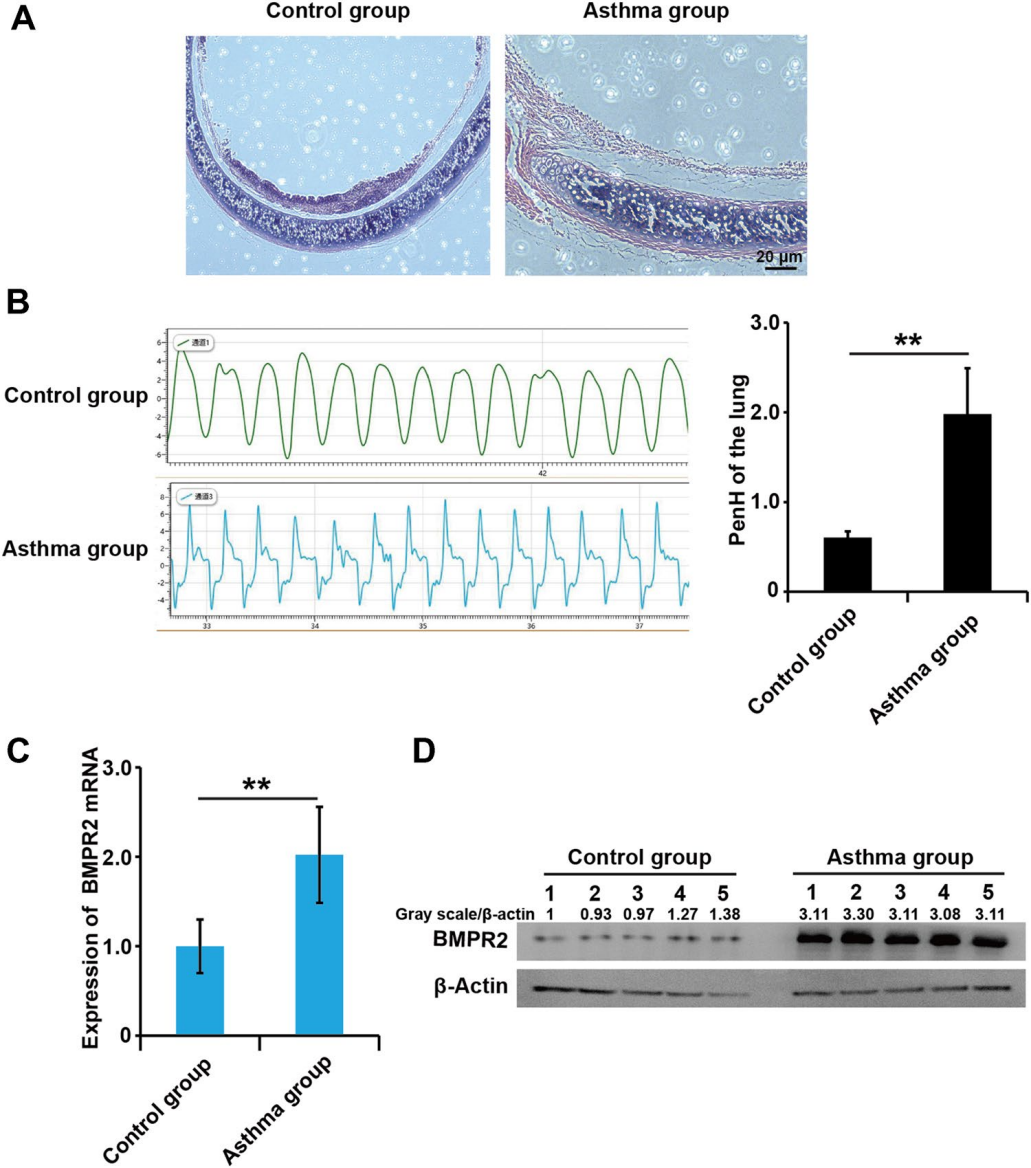
BMPR-II were upregulated in the airway of allergic asthma rats. **A** HE staining of lung tissue and bronchial wall in rats, ×40. The picture shows the lung tissue and airway wall constructions in control group (Left) and asthma group (Right). **B** The enhanced pause (Penh) measured by WBP whole body plethysmography system. Reference range of PENH index: Normal: PENH < 0.82; lung injury: PENH > 0.82; pulmonary fibrosis: PENH > 1.5. **C** and **D** The mRNA and protein levels of BMPR-II in airway smooth muscle layer from non-asthma and asthma model rats. was detected by qPCR assay. β-Actin was used as internal control. All samples were replicated in triplicate for qPCR. Data are expressed as the mean ± standard deviation. **P < 0.01 was considered to indicate a statistically difference compared with the control

The numbers of mucosal and submucosal inflammatory cells in lung tissue stimulated by ovalbumin were significantly augmented compared to the control group. Meanwhile, the airway wall and airway smooth muscle layer in asthma rats were markedly thicker than in the control group (Fig. 1A). Meanwhile, the enhanced pause (Penh) of the rats increased in in asthma rats (Fig. 1B). Overall, these results demonstrated that this study successfully constructed the asthma rat model. To explore the role of BMPR-II in asthma, we detected expression of BMPR-II in airway smooth muscle layer from non-asthma and asthma model rats. qPCR results demonstrated that mRNA expression level of BMPR-II was significantly upregulated in asthma model rats compared to non-asthma rats (Fig. 1C). Similar to the mRNA expression patterns, western blots results showed that the protein expression level of BMPR-II were significantly up-regulated in asthma model rats compared to non-asthma rats (Fig. 1D).

Overall, these results suggested that BMPR-II were involved in the progression of asthma.

### Growth, migration and intracellular Ca^2+^ levels of ASMCs were significantly increased in ASMCs from asthmatic rats

**Fig. 2:**
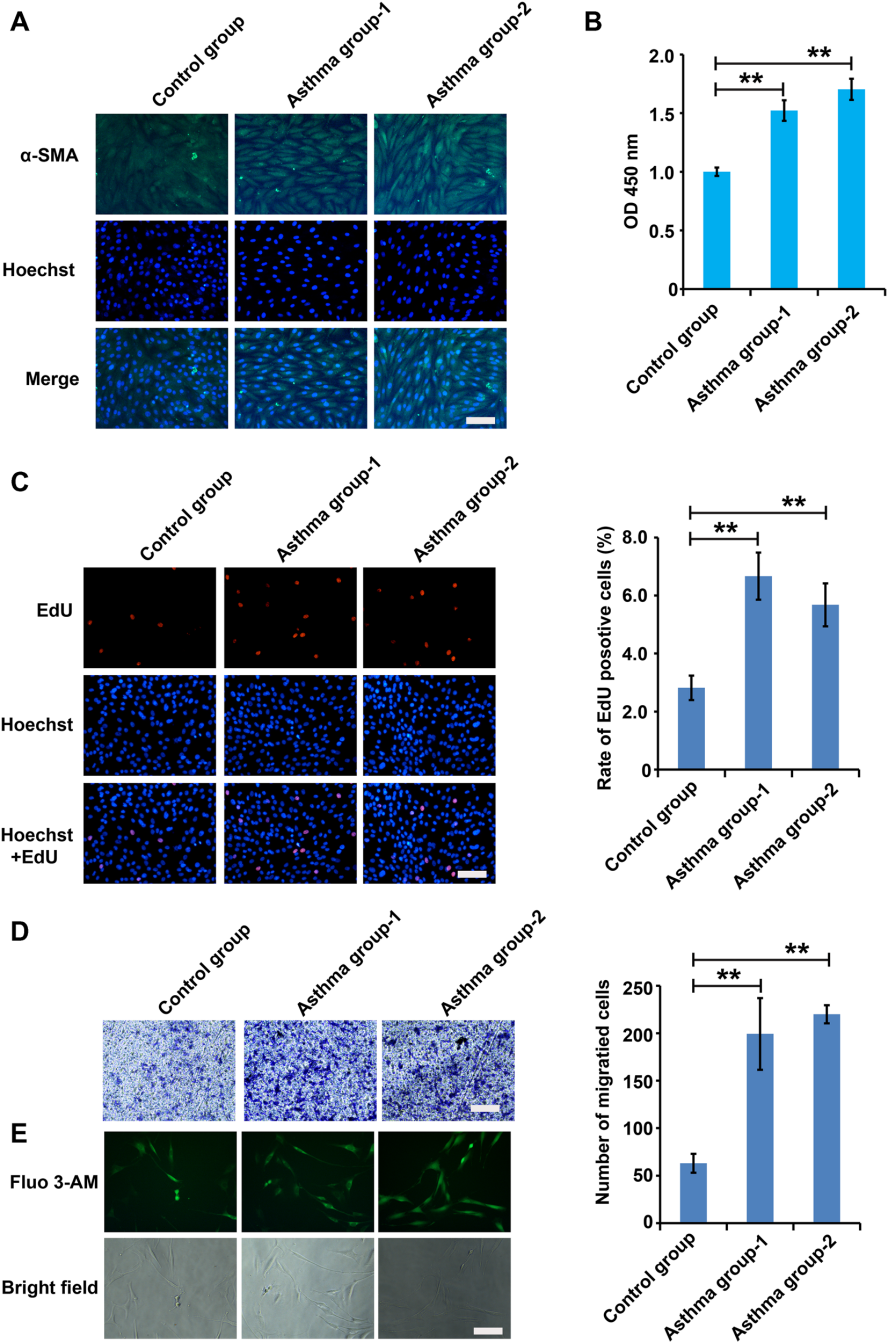
Growth, migration and intracellular Ca^2+^ levels of ASMCs were significantly increased in ASMCs from asthmatic rats. **A** Representative immunofluorescent stained cells from trachea of wistar rats in the control group and asthma group. Upper: wistar rats ASM cells under the luminescence microscope following immunofluorescent identification by α-SMA staining. Magnification ×400. **B** The viability of ASMCs was detected using CCK8 analysis. **C** The proliferation of ASMCs was detected using EdU analysis. **D** The migration of ASMCs was detected using Transwell analysis. **E** The concentration of intracellular calcium was detected using Fluo-3AM assay. For **A**–**E**, cells were divided into three groups: two different ASMCs isolated from asthmatic rats and one ASMCs isolated from normal rats. All samples were replicated in triplicate for qPCR, CCK8, EdU and migration. Data are expressed as the mean ± standard deviation. There was a significant difference between the two groups. **P < 0.01 versus the control group

Then ASMCs were isolated and smooth muscle α-actin (α-SMA) immunofluorescence was performed to identify ASMCs in asthmatic rats. These results in the allergic asthma group are shown in Fig. 2A. We observed that subcultured cells presented as primarily as fusiform and sometimes as polygonal arranged neatly with round nuclei located in the centre, and ASMCs was α-SMA positive.

As shown in Fig. 2B–D, the cell viability, proliferation and migration were enhanced in of ASMCs isolated from asthmatic rats. Meanwhile, ASMCs isolated from asthmatic rats displayed markedly increased Ca^2+^ concentrations compared to control cells (Fig. 2E), suggested that this study successfully constructed the cell model of asthma.

### Silencing of BMPR-II inhibited the cell viability, proliferation, migration capacity, and intracellular Ca ^2+^ concentration in asthmatic rats ASMCs

**Fig. 3:**
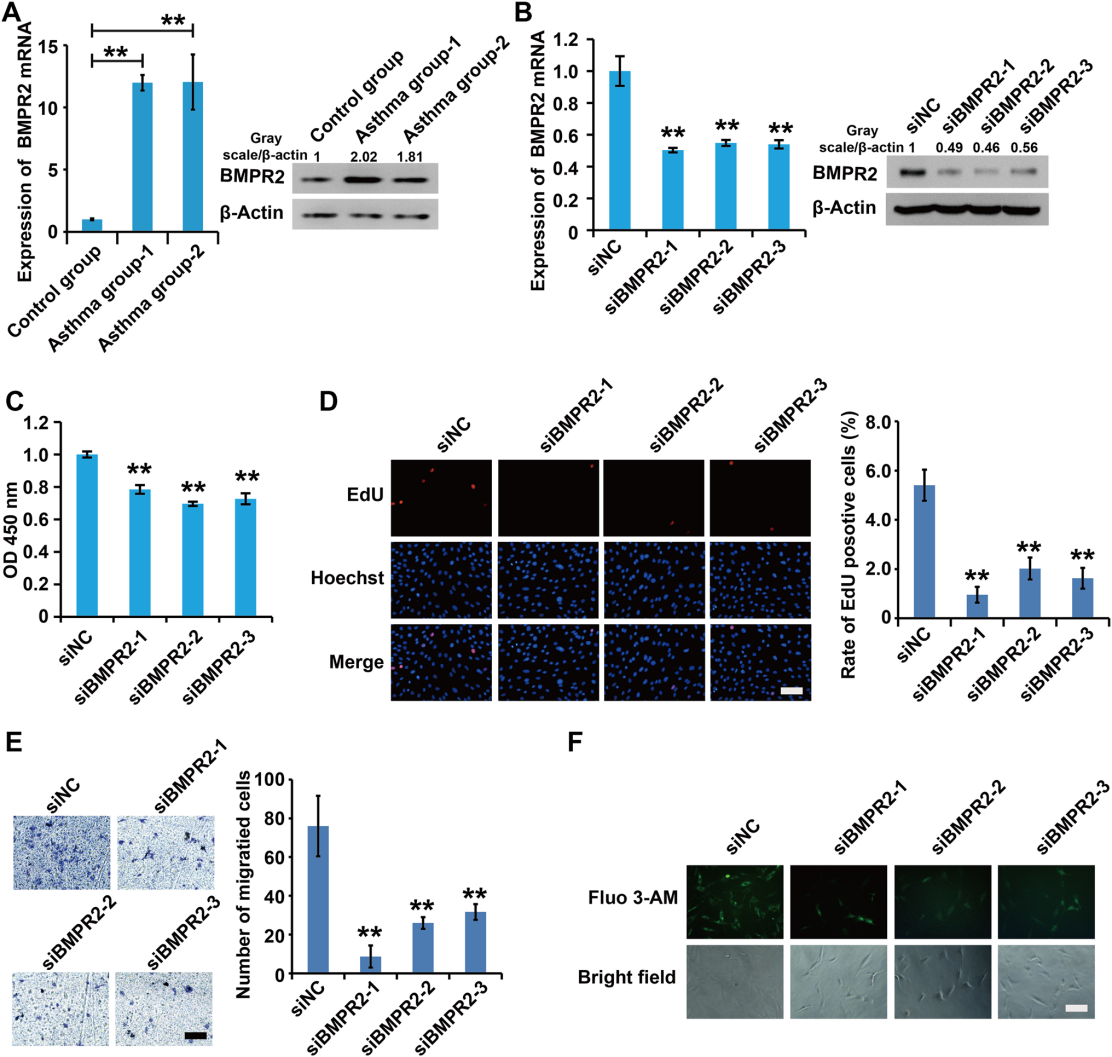
Silencing of BMPR-II inhibited the cell viability, proliferation, migration capacity, and intracellular Ca^2+^ concentration in asthmatic rats ASMCs. **A** The mRNA and protein levels of BMPR-II in ASMCs were detected by qPCR and western blots assay in asthma group and control group. **B** qPCR and western blot assay analysis of the validity of siBMPR-II. **C** The viability of ASMCs was detected using CCK8 analysis. **D** The proliferation of ASMCs was detected using EdU analysis. **E** The migration of ASMCs was detected using Transwell analysis. **F** The concentration of intracellular calcium was detected using Fluo-3AM assay. For **B**–**F** cells were transfected with siBMPR2s and control siRNAs for 48 h. All samples were replicated in triplicate for qPCR, CCK8, EdU and migration. Data are expressed as the mean ± standard deviation. There was a significant difference between the two groups. **P < 0.01 versus the control group

To explore the role of BMPR-II in asthma, we detected expression of BMPR-II in ASMCs from non-asthma and asthma model rats. qPCR and western blots results demonstrated that expression levels of BMPR-II, were significantly upregulated in ASMCs from asthma model rats compared to non-asthma rats (Fig. 3A). These results imply that high expression of BMPR-II, might be associated with the progression of asthma.

To further examine the effects of BMPR-II on the bioactivity of asthmatic ASMCs, small interfering RNA against BMPR-II (siBMPR-II) and their control siRNAs (siNC) were designed and transfected into ASMCs, respectively. First, qPCR and western blot analysis displayed that all three siBMPR-IIs efficiently inhibited BMPR-II expression in asthmatic ASMCs, and siBMPR-II-2 was chosen for subsequent experiments (Fig. 3B). CCK8, EdU, migration and Fluo-4AM analysis demonstrated that silencing siBMPR-II reduced cell viability, inhibited cell proliferation, migration and attenuated intracellular Ca^2+^ concentration in ASMCs (Fig. 3C–F).

Collectively, these results demonstrated that repression of BMPR-IIinhibited cell viability, proliferation, migration capacity, and intracellular Ca^2+^ concentration in asthmatic rat ASMCs.

### Silencing of BMPR-II inhibited the SMAD3 expression and SMAD3 promoted the cell viability, proliferation, migration capacity, and intracellular Ca ^2+^ concentration in asthmatic rats ASMCs

**Fig. 4:**
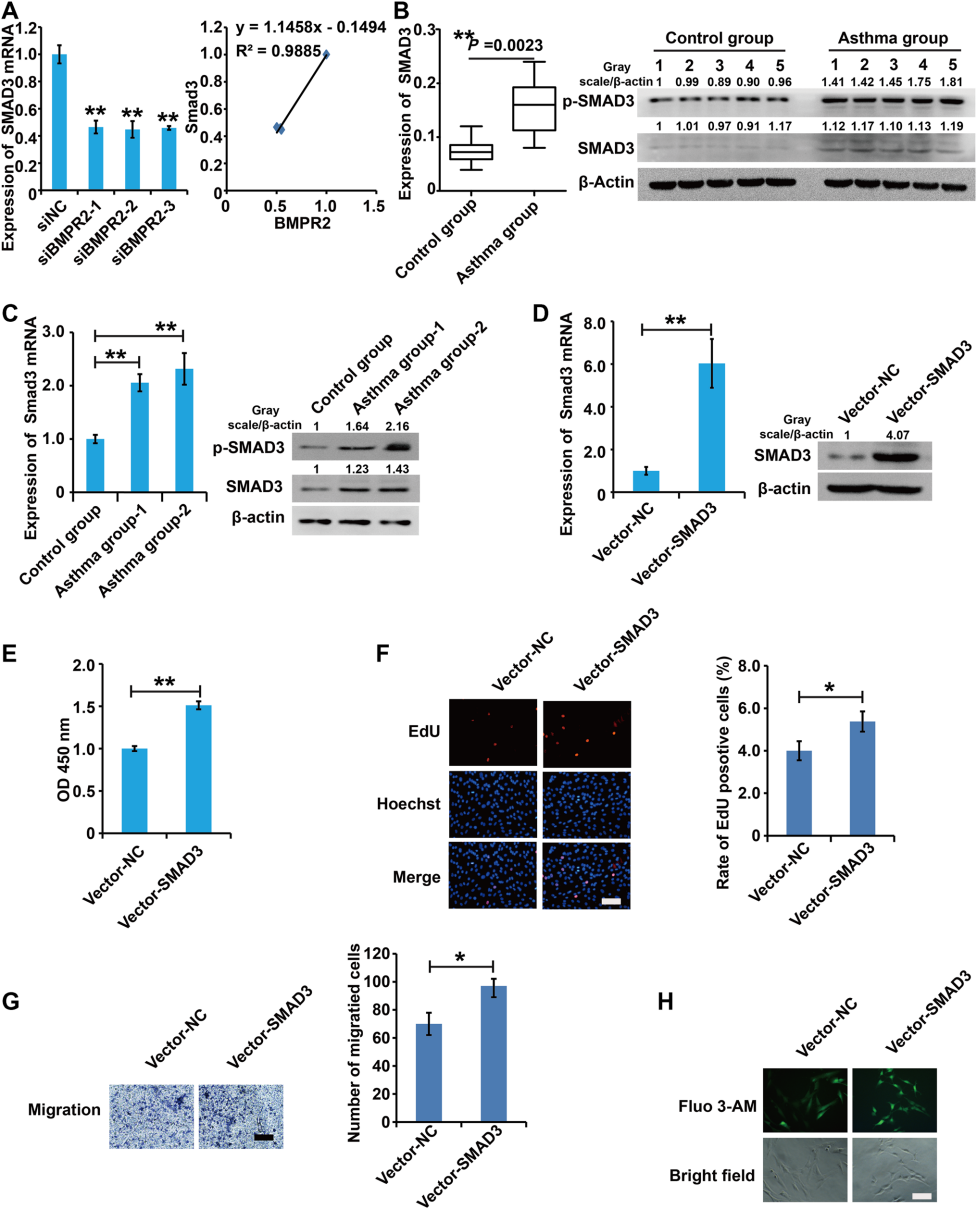
Silencing of BMPR-II inhibited the SMAD3 expression and SMAD3 promoted the cell viability, proliferation, migration capacity, and intracellular Ca^2+^ concentration in asthmatic rats ASMCs. **A** qPCR and western blot assay analysis of the SMAD3 expression in cells transfected with siBMPR2s and control siRNAs for 48 h. **B** qPCR and western blot assay analysis of the expression of Smad3 in airway smooth muscle layer from non-asthma and asthma model rats. **C** qPCR and western blot assay analysis of the expression of Smad3 in ASMCs from non-asthma and asthma model rats. **D** qPCR and western blot assay analysis of the validity of vector-SMAD3. **E** The viability of ASMCs was detected using CCK8 analysis. **F** The proliferation of ASMCs was detected using EdU analysis. **G** The migration of ASMCs was detected using Transwell analysis. **H** The concentration of intracellular calcium was detected using Fluo-3AM assay. For **D**–**H**, cells were transfected with vector-SMAD3s and control vectors for 48 h. All samples were replicated in triplicate for qPCR, CCK8, EdU and migration. Data are expressed as the mean ± standard deviation. There was a significant difference between the two groups. **P < 0.01 versus the control group

Previous study demonstrated that BMPR-II affected the SMAD3 expression in pulmonary arterial hypertension [17]. The results of qPCR showed that silencing of BMPR-II inhibited the SMAD3 expression in asthmatic rat ASMCs (Fig. 4A).

To explore the role of BMPR-II in asthma, we detected expression of BMPR-II in airway smooth muscle layer and ASMCs from non-asthma and asthma model rats. qPCR and western blots results demonstrated that mRNA and protein expression levels of SMAD3 and its phosphorylation type pSmad3 were significantly upregulated in airway smooth muscle layer and ASMCs from asthma model rats compared to non-asthma rats (Fig. 4B, C). Overall, these results suggested that BMPR-II were involved in the progression of asthma.

To further examine the effects of SMAD3 on the bioactivity of asthmatic ASMCs, ectopic expression of SMAD3 (Vector-SMAD3) and their control plasmids (Vector-NC) were transfected into ASMCs, respectively. First, qPCR and western blot analysis displayed that Vector-SMAD3 efficiently elevated the Smad3 expression in asthmatic ASMCs (Fig. 4D). CCK8, EdU, migration and Fluo-4AM analysis demonstrated that upregualtion of SMAD3 promoted the cell viability, inhibited cell proliferation, migration and attenuated intracellular Ca^2+^ concentration in ASMCs (Fig. 4E–H).

Collectively, these results demonstrated that SMAD3 promoyed the cell viability, proliferation, migration capacity, and intracellular Ca^2+^ concentration in asthmatic rat ASMCs.

### SMAD3 interacted with MRTF and silencing of MRTF inhibited the cell viability, proliferation, migration capacity, and intracellular Ca ^2+^ concentration in asthmatic rats ASMCs

**Fig. 5:**
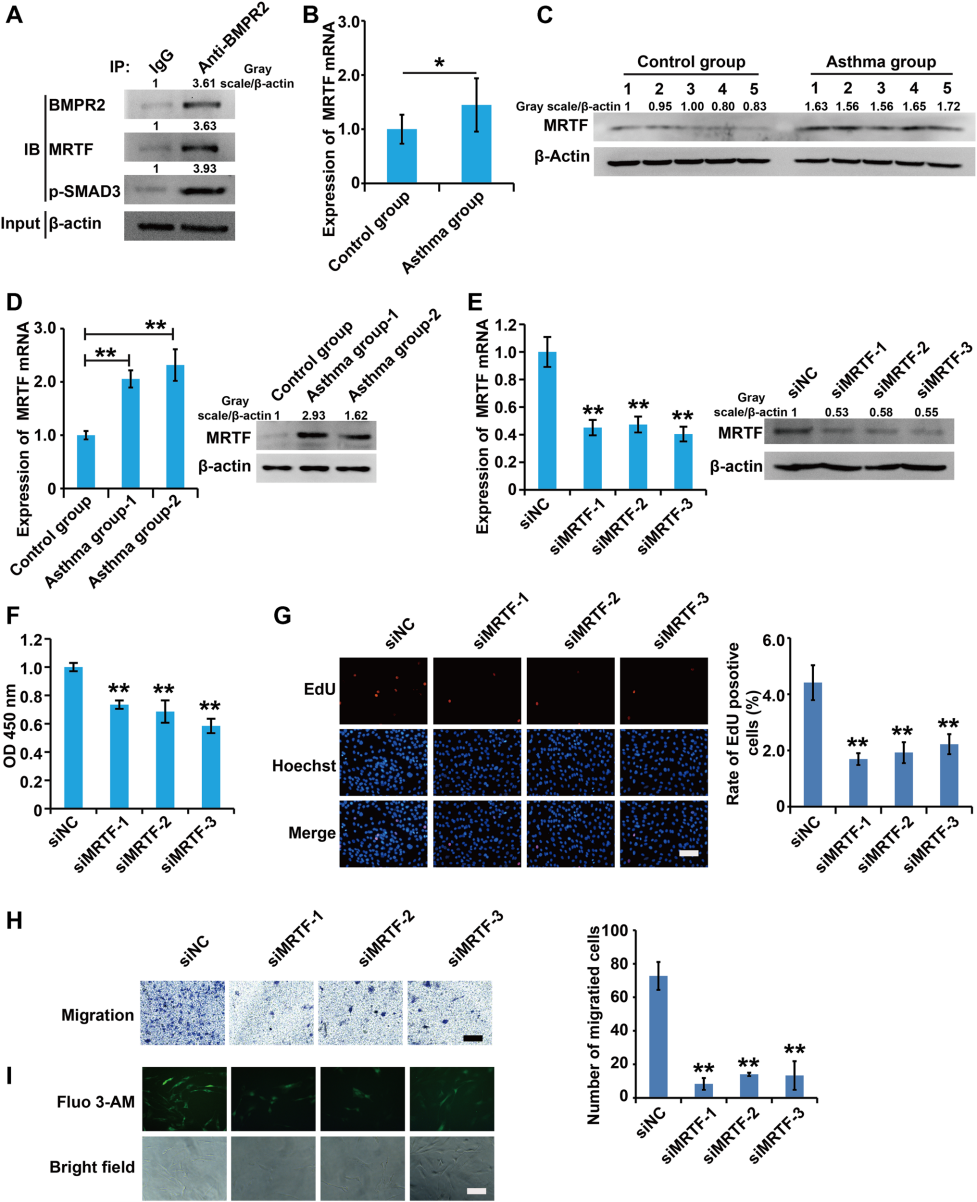
SMAD3 interacted with MRTF and silencing of MRTF inhibited the cell viability, proliferation, migration capacity, and intracellular Ca^2+^ concentration in asthmatic rats ASMCs. **A** Immunoprecipitation assay measured the interaction between SMAD3 and MRTF. Control immunoprecipitation was performed using pre-immune IgG. IP represents immunoprecipitation and IB indicates immunoblot. **B**–**D** qPCR and western blot assay analysis of the expression of Smad3 in airway smooth muscle layer and ASMCs from non-asthma and asthma model rats. **E** qPCR and western blot assay analysis of the validity of siMRTF. **F** The viability of ASMCs was detected using CCK8 analysis. **G** The proliferation of ASMCs was detected using EdU analysis. **H** The migration of ASMCs was detected using Transwell analysis. **I** The concentration of intracellular calcium was detected using Fluo-3AM assay. For **E**–**I**, cells were transfected with siMRTFs and control siRNAs for 48 h. All samples were replicated in triplicate for qPCR, CCK8, EdU and migration. Data are expressed as the mean ± standard deviation. There was a significant difference between the two groups. **P < 0.01 versus the control group

Previous studies have shown there is a direct link between SMAD3 and MRTF in asthma. Thus, we examined whether SMAD3 and MRTF form a complex in asthmatic rat ASMCs. To answer this question, we performed co-immunoprecipitation analysis, and results demonstrated that the anti-SMAD3 antibody could pull down MRTF, uncovering interaction between SMAD3 and MRTF (Fig. 5A).

To explore the role of MRTF in asthma, we detected expression of MRTF in airway smooth muscle layer and ASMCs from non-asthma and asthma model rats. qPCR and western blots results demonstrated that mRNA and protein expression levels of MRTF were significantly upregulated in airway smooth muscle layer and ASMCs from asthma model rats compared to non-asthma rats (Fig. 5B–D). Overall, these results suggested that MRTF were involved in the progression of asthma.

To further examine the effects of MRTF on the bioactivity of asthmatic ASMCs, small interfering RNA against siMRTF (siMRTF) and their control siRNAs (siNC), were designed and transfected into ASMCs, respectively. First, qPCR and western blot analysis displayed that all three siMRTFs efficiently inhibited MRTF expression in asthmatic ASMCs, and siMRTF-2 was chosen for subsequent experiments (Fig. 5E). CCK8, EdU, migration and Fluo-4AM analysis demonstrated that silencing siMRTF reduced cell viability, inhibited cell proliferation, migration and attenuated intracellular Ca^2+^ concentration in ASMCs (Fig. 5F–I).

Collectively, these results demonstrated that repression of MRTF inhibited cell viability, proliferation, migration capacity, and intracellular Ca^2+^ concentration in asthmatic rat ASMCs.

### Effects of the BMPR-II-SMAD3/MRTF complex on proliferation and migration capacity

**Fig. 6:**
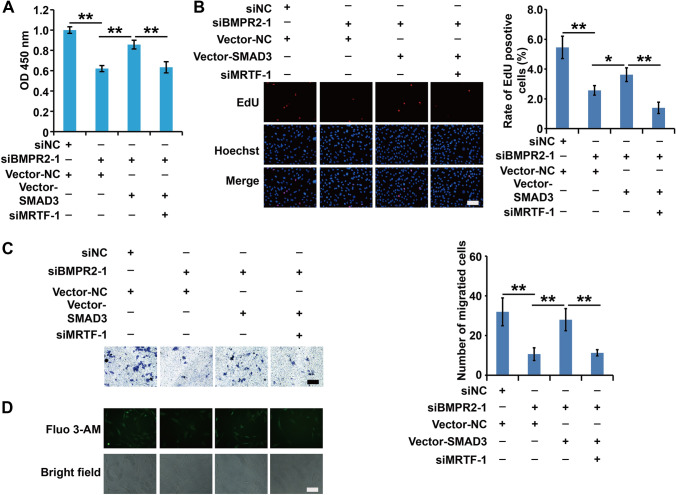
The effect of BMPR-II-SMAD3/MRTF signaling on the viability and migration of ASMCs. **A** The viability of ASMCs with different treatment was detected using CCK8 analysis. **B** The proliferation of ASMCs with different treatment was detected using EdU analysis. **C** The migration of ASMCs with different treatment was detected using Transwell analysis. **D** The concentration of intracellular calcium in ASMCs with different treatment was detected using Fluo-3AM assay. For **A**–**D**, cells were divided into four groups: siNC + pcDNA-NC, siBMPR-II + pcDNA-NC, siBMPR-II + pcDNA-SMAD3 and siBMPR-II + pcDNA-SMAD3 + siMRTF groups. All samples were replicated in triplicate for qPCR, CCK8, EdU and migration. Data are expressed as the mean ± standard deviation. There was a significant difference between the two groups. **P < 0.01 versus the control group

To confirm the physical or functional interaction among BMPR-II, SMAD3, and MRTF, we subsequently performed the following experiments. First, we divided ASMCs into four groups: siNC + pcDNA-NC, siBMPR-II + pcDNA-NC, siBMPR-II + pcDNA-SMAD3 and siBMPR-II + pcDNA-SMAD3 + siMRTF groups. CCK8, migration and Fluo-4AM analysis revealed that any disruption to the BMPR-II-SMAD3/MRTF complex affected cell viability, migration and intracellular Ca^2+^ concentration in ASMCs (Fig. 6A–C). Collectively, these results demonstrate that the BMPR-II-SMAD3/MRTF complex affects cell viability, migration capacity, and intracellular Ca^2+^ concentration in asthmatic rat ASMCs.

## Discussion

Allergic asthma, an inflammatory respiratory disease, is defined as the inevitable result of chronic airway inflammation, airway hyperresponsiveness, and airway remodelling [20, 21]. Airway remodelling predominantly refers to changes in the airway wall components, which include epithelial shedding, fibroblast accumulation, peribronchial interstitial tissue changes, proliferation of the bronchovascular system, and increased smooth muscle mass [22]. These changes lead to airway stenosis and irreversible airway obstruction, which is the pivotal feature of recurrent asthma attacks [23]. ASM is the major tissue regulating airway lumen and predominantly mediating airway stenosis through contracting of airway smooth muscle, which were a critical target of historic asthma treatments [24]. Airway hyperresponsiveness is characteristic of strong contraction of the ASMCs when stimulated by a bronchoconstrictor [4]. However, not all patients with asthma exhibit an increase in ASM, and the pathological mechanism of ASM mediating the airway diameter remains unclear [25]. In recent years, increasing studies have shown that ASM remodelling occurs before airway inflammation, and ASMCs may represent the primary contributor to airway inflammation [26]. Airway inflammation promotes ASMC contraction and proliferation, whereas ASM remodelling aggravates airway inflammation by secreting a series of cytokines, forming a vicious cycle [27]. Current therapeutic drugs comprise corticosteroids and bronchodilators [28]. However, there are still patients who experience severe refractory asthma and develop resistance to these therapies [29]. Hence, it is imperative for us to identify new targets and to develop novel pharmacological interventions.

Recent studies have found that BMPR-II, SMAD3, and MRTF are all expressed in ASMCs in asthma models [11, 12, 15]. The contractility of ASMCs is largely dependent on intracellular Ca^2+^ concentrations; in addition, intrinsic increases in Ca^2+^ levels contribute to ASMC proliferation to some extent [30]. Moreover, prior studies demonstrated that instant Ca^2+^ transiently plays an important role in regulating cellular processes, such as proliferation and signal transduction [30]. Therefore, it is crucial to explore mechanisms for balancing the releasing of intracellular Ca^2+^ storage and extracellular Ca^2+^ entry to maintain Ca^2+^ homeostasis. Our experiments first revealed that BMPR-II (2.02 times in asthma vs. control groups), SMAD3 (2.14 times in asthma vs. control groups), and MRTF (1.45 times in asthma vs. control groups) are highly expressed in ASMCs in our asthma model. Furthermore, we found that inhibition of BMPR-II, SMAD3, and MRTF using siRNAs decreased proliferation and migration of ASMCs. Next, we confirmed that BMPR-II, SMAD3, and MRTF form a complex in our atopic asthma model, as evidenced by immunoprecipitation assays. All three molecules in this complex affect intracellular Ca^2+^ levels. Together with previous studies, these results led us to hypothesize that this complex may play a crucial role in the regulation of cellular bioactivities, and the interaction between these three molecules may modulate contraction and proliferation of ASMCs through inducing the release of Ca^2+^. Furthermore, all three factors were indispensable to the function of the complex.

To explore this interaction and specific functional mechanisms involved, we performed several additional experiments. First, we knocked down BMPR-II in asthmatic ASMCs and observed decreased proliferation and migration rates of ASMCs. Since all three factors were highly expressed in ASMCs and BMPR-II was inhibited by siBMPR-II, we subsequently selected pcDNA SMAD3 to overexpress SMAD3 to detect the interaction between SMAD3 and BMPR-II. As expected, we found that ASMCs treated with pcDNA SMAD3 exhibited increased proliferation and migration compared to the cells with BMPR-II inhibition. When cells were treated with siMRTF, cell viability, proliferatiom and migration decreased compared to cells in the siBMPR-II-pcDNA SMAD3 group. However, there was no significant difference compared to ASMCs in the siBMPR-II group. Taken together, these findings support a physical and functional interaction among BMPR-II, SMAD3, and MRTF, which may involve regulation of proliferation and migration in ASMCs through mediating intracellular Ca^2+^ concentrations. However, this specific mechanism needs to be further studied.

Thus, this study displays the functional complex, BMPR-II-SMAD3/MRTF signalling pathway, and further, revealed their huge role in the process of ASM remodelling, including the cell growth, migration and intracellular Ca^2+^ concentration and thus, indicates a potential of the complex in construction of the novel treatment for asthma.

## Data Availability

Data and material are available after required.
